# Characterizing network dynamics of online hate communities around the COVID-19 pandemic

**DOI:** 10.1007/s41109-021-00362-x

**Published:** 2021-03-05

**Authors:** Joshua Uyheng, Kathleen M. Carley

**Affiliations:** CASOS Center, Institute for Software Research, Carnegie Mellon University, 5000 Forbes Ave, Pittsburgh, PA USA

**Keywords:** Hate speech, Constructural theory, Dynamic network analysis, Infodemic, COVID-19 pandemic

## Abstract

Hate speech has long posed a serious problem for the integrity of digital platforms. Although significant progress has been made in identifying hate speech in its various forms, prevailing computational approaches have tended to consider it in isolation from the community-based contexts in which it spreads. In this paper, we propose a dynamic network framework to characterize hate communities, focusing on Twitter conversations related to COVID-19 in the United States and the Philippines. While average hate scores remain fairly consistent over time, hate communities grow increasingly organized in March, then slowly disperse in the succeeding months. This pattern is robust to fluctuations in the number of network clusters and average cluster size. Infodemiological analysis demonstrates that in both countries, the spread of hate speech around COVID-19 features similar reproduction rates as other COVID-19 information on Twitter, with spikes in hate speech generation at time points with highest community-level organization of hate speech. Identity analysis further reveals that hate in the US initially targets political figures, then grows predominantly racially charged; in the Philippines, targets of hate consistently remain political over time. Finally, we demonstrate that higher levels of community hate are consistently associated with smaller, more isolated, and highly hierarchical network clusters across both contexts. This suggests potentially shared structural conditions for the effective spread of hate speech in online communities even when functionally targeting distinct identity groups. Our findings bear theoretical and methodological implications for the scientific study of hate speech and understanding the pandemic’s broader societal impacts both online and offline.

## Introduction

It has become a matter of broad consensus that COVID-19 cannot be seen solely as a public health problem, but also as a crisis involving deep social and psychological issues (Van Bavel et al. 2020). As nations grapple with the operational challenges of mounting robust medical and economic programs to curb outbreaks, the global pandemic has also stoked heated social divisions (Chiriboga et al. 2020; Martinez-Juarez et al. 2020).

One significant context in which such conflicts take place is the digital sphere (Gallotti et al. 2020; Starbird et al. 2019). Over the past decade, social media has democratized public discourse on an unprecedented scale. But the open flow of information in cyberspace has also enabled widespread abuse (Beskow and Carley 2019; Carley et al. 2018a). Defined broadly as a form of negative language targeting specific identities, online hate speech has been flagged as a serious concern across popular digital platforms (Davidson et al. 2017; Warner and Hirschberg 2012). Recent algorithmic developments suggest that, although a non-trivial task, computational approaches to hate speech detection may be used to identify online toxicity in large-scale social media conversations (Alorainy et al. 2019; Badjatiya et al. 2017; ElSherief et al. 2018a; Fortuna and Nunes 2018).

Past studies likewise demonstrate how hate speech not only inhibits civil discourse online, but also potentially links to real-world consequences of violence and discrimination, especially against marginalized groups (Awan and Zempi 2016; Johnson et al. 2019). In the context of the pandemic, emerging reports characterize the eruption of online hate along fraught racial lines (Stechemesser et al. 2020; Ziems et al. 2020). Recognizing the complex interplay of socio-technical factors related to digital communication (Geschke et al. 2019; Perra and Rocha 2019), we posit the importance of examining hate speech not only in terms of its linguistic prevalence, but also in terms of its community dynamics (Bilewicz and Soral 2020; Johnson et al. 2019; Waqas et al. 2019).

Informed by constructural theory, we extend prevailing computational approaches for hate speech identification by attending to its socially situated spread within evolving social networks (Carley 1991, 1989, 1995; Joseph et al. 2014). Examining Twitter conversations about the pandemic in the Philippines and the United States, we formulate methods for characterizing online hate speech and the communities which propagate it over time (Uyheng et al. 2019). More specifically, we probe how network clusters with higher levels of hate speech systematically differ from others both in terms of *structural* (e.g., density, echo chambers) and *functional* (e.g., targeting of specific identities) features (Crenshaw 1990; Joseph et al. 2016; Kim 2020). Taken together, our findings bear theoretical and methodological implications for the scientific study of hate speech and understanding the pandemic’s broader societal impacts both online and offline (Luengo-Oroz et al. 2020; Pohjonen and Udupa 2017; Reicher and Stott 2020).

To orient the reader, the succeeding sections of this paper engage prior related work as follows. First, we offer a brief overview of the problem of hate speech as both a computational and social issue, reviewing some general approaches for understanding and detecting hate speech on digital platforms. Second, we look at issues of ‘infodemics’ during the COVID-19 pandemic. Noting the complexity of issues surrounding information flow and social relations during the pandemic, we highlight the need for integrative views of online conflicts during crisis. Finally, drawing on constructural theory, we consider ways of addressing extant conceptual and methodological gaps. Building on these foundations, we therefore ask: How does hate speech evolve within online communities in the context of the COVID-19 pandemic?

## Related work

### The problem of online hate speech

Understanding online hate speech represents a crucial, multidisciplinary challenge (Pohjonen and Udupa 2017; Warner and Hirschberg 2012). Seminal work distinguishes hate speech from regular speech on social media based on its targeted attack of specific identities; from this perspective, it is also different from offensive speech which merely uses uncouth or inappropriate language (Davidson et al. 2017). Social scientific research empirically highlights the negative effects of online hate speech on individual psychological well-being and wider intergroup relations. Exposure to exclusionary hate speech bears negative emotional consequences for marginalized identities (Leader et al. 2009; Saha et al. 2019). As populations consume online content with increased levels of toxicity, higher levels of prejudice may be observed in line with growing experiences of desensitization (Soral et al. 2018). In the long term, widespread hate speech likewise contributes to greater likelihood of radicalization against vulnerable, targeted groups (Bilewicz and Soral 2020).

From a computational perspective, identifying online hate speech in large-scale conversations with scalable tools thus comprises an important task. Recent advances in machine learning and natural language processing have resulted in important progress with respect to automated hate speech detection. Cutting-edge techniques for identifying hate speech have successfully deployed deep-learning methods to account for the complex linguistic features which characterize toxic utterances online (Badjatiya et al. 2017; Fortuna and Nunes 2018). More theoretically motivated work has similarly developed lexicon-based or embedding-based approaches, with unique applications to examine the targets of hate speech (Alorainy et al. 2019; ElSherief et al. 2018a, b), or to probe the unique ways online populations have countered hate speech (Chung et al. 2019; Mathew et al. 2019).

Viewing hate speech from the lens of both the computational and social sciences bears important implications for addressing its spread in both online and offline contexts (Carley et al. 2018a). Whereas algorithmic tools may help flag particular instances of hate speech (Burnap and Williams 2015), policy responses require an understanding of the contextual conditions which facilitate its spread. These broadly include the sources and targets of hate speech, the idiosyncrasies of particular social media platforms, and wider aspects of society at large (Jardine 2019; Pater et al. 2016; Starbird et al. 2019).

### Hate speech as infodemic: from texts to communities

Many of these issues have been brought to sharp relief in the context of the ongoing COVID-19 pandemic. The present global climate of uncertainty and fear has fomented intense public need for information and deepened societal fissures (Chiriboga et al. 2020; Martinez-Juarez et al. 2020). Running parallel to the networked epidemics of COVID-19 itself (Hâncean et al. 2020), these conditions have contributed to a so-called ‘infodemic’ on cyberspace—an overabundance of information of extremely variable quality, communicated at unprecedented rates, with numerous potentially adverse consequences (Cinelli et al. 2020; Zarocostas 2020).

Online hate speech features as one major aspect of this infodemic. Research finds that racism against Asian populations has notably spiked due to associations made between the virus and its originating infections in China (Stechemesser et al. 2020; Ziems et al. 2020). Some scholars point to the role of political leaders in promoting inclusive social order or exclusionary social disorder when communicating with their followers (Cohen 2020; Reicher and Stott 2020). Others also point to how the inherent features of cyberspace may contribute to the formation of hate speech communities and the viral spread of toxic content related to COVID-19 (Johnson et al. 2019; Kim 2020).

Both enduring and burgeoning concerns around the pandemic thus point to the need for understanding online hate speech beyond a purely linguistic perspective. As an infodemic co-occurring with the COVID-19 pandemic, hate speech must also be considered in terms of its social spreading dynamics. However, prevailing computational scholarship concentrates on the classification of hate speech as a static, textual phenomenon, without explicit consideration for the communicative contexts in which it proliferates.

Hence, beyond the prevalence of hate speech, we suggest that it is important to focus on hate communities. This bears several implications for our proposed approach. First, existing studies tend to operate on a single functional view of (racist) hate during the pandemic. However, not all communities engage in the same form of hate (Crenshaw 1990; Priante et al. 2016). Furthermore, we consider the structural features of hate communities. Past attention to the communicative conditions which characterize the spread of online toxicity largely remains limited (Kim 2020). Finally, we note that the primary focus in scholarly work has largely been on hate speech in Western or English-speaking contexts against Asian populations (Henrich et al. 2010). While valuable, these studies do not address the global scale of both the pandemic and its universal strain on unequal societies worldwide (Gosling et al. 2010; Uyheng and Carley 2019).

### A constructural view of dynamic hate networks

To address these conceptual and methodological gaps, we draw insights from the social scientific theory of constructuralism. Constructural theory understands evolving social phenomena through the dynamic interplay of cognitive, interactional, and technological factors (Carley 1989, 1991, 1995). Under this framework, individuals possess a particular set of beliefs. The informational properties of individuals shape preferences for interactions with other individuals. As social interactions progress, individuals update their beliefs, which in turn reshape interaction preferences. Taken together, these factors collectively contribute to the structural properties of large-scale social networks. Social networks, moreover, are seen to co-evolve with knowledge structures or cultural norms (Joseph et al. 2014, 2016). In other words, as patterns of interconnection between agents shift, so do the relationships between the ideas they exchange.

Viewed from this perspective, online hate speech around the pandemic may be seen as a dynamic, community-based phenomenon, with unique properties in distinct political and cultural settings. In terms of the content of hate speech, we posit that patterns of toxicity and aggression depend on how particular identities are targeted. Hence, for instance, hate toward certain identities may take on different forms of abuse over time, or combine with hate toward other identities. We also hypothesize that the structural features of social networks, which are linked to possibilities and constraints for information flow, are predictive of hate speech prevalence. From a network perspective, we surmise that hate may spread more successfully in communities that are more isolated from the broader conversation. Communities with more hateful opinion leaders might also feature higher levels of overall hate speech.

### Contexts of study

To investigate these propositions in this work, we consider comparative analysis of the contexts of the United States and the Philippines. A comparative approach enables us to determine shared, as well as contextually unique, dynamics of hate speech across distinct geopolitical settings (Miller and Vaccari 2020).

Both the US and the Philippines have faced particular challenges in curbing COVID-19 outbreaks, with the US confronting the largest cumulative number of confirmed cases in the world, and the Philippines the largest for Southeast Asia. Additionally, both the US (Rutledge 2020) and the Philippines (Uyheng and Montiel 2020b) have been noted for their contexts of political polarization under populist leadership. These factors contribute to additional difficulties in pandemic management, but also potentially constitute conditions for exacerbated social conflicts (Reicher and Stott 2020).

But while these two countries share common political features, they are also unique contexts for the spread of hate speech. For instance, we note that US-specific concerns with racism have vastly outlived the COVID-19 pandemic (Abramowitz and McCoy 2019; Crenshaw 1990). Hate speech in the US may thus hinge on historical racial divisions on top of more recent spikes in international tensions with China (Beskow and Carley 2020; Ziems et al. 2020). On the other hand, the Philippines’ geographic proximity to China, and its recent history of territorial disputes with the Asian superpower, may also push particular forms of sinophobic discourse on digital platforms (Montiel et al. 2019; Ong et al. 2019).

We note that while these past studies do not strictly suggest predictive hypotheses for the dynamics of hate speech examined here, they contextualize potential reasons for the emergence of online hate speech during the COVID-19 pandemic, especially with respect to racial groups or political figures. Other countries may feature distinct dynamics throughout the course of the pandemic as shaped by their own local societal conflicts.

### Contributions of this work

In adopting a constructural view, we introduce an integrative computational framework for understanding online hate communities during the pandemic. Going beyond a purely textual analysis, we compare the network dynamics of hate speech across distinct geopolitical settings. More specifically, this paper therefore stakes the following contributions to the literature:

1. we associate the spread of online hate speech with structural features of hate communities;
2. we explicitly examine diverse forms and targets of online hate speech; and
3. we perform comparative analysis across two unique political and cultural contexts hit hard by the COVID-19 pandemic.

## Data and methods

### Data collection

Online conversations around the COVID-19 pandemic were collected using Twitter’s REST application programming interface (API). Search terms were specified to obtain tweets related to the pandemic in the Philippines and the US. Both countries respectively used ‘#COVID19PH’ and ‘#COVID19US’ as localized hashtags for discussing the disease. Data collection for this study lasted from March 5 to May 19 of 2020 over a period of 75 days in total. In the US, this end date corresponded to a week before the #BlackLivesMatter protests. Each dataset was stored in JSON format with user metadata, tweet metadata, and data on the interactions between users in the form of retweets, replies, quotes, and mentions. Data for each tweet also contained information about the hashtags and URLs it used.

At the end of data collection, a total of 15 million tweets representing 1 million users was collected for the Philippines. For the US, a dataset of 12 million tweets representing 1.6 million users was obtained. Given that the US has three times the population of the Philippines, we hypothesize that the discrepancy in dataset size between the two countries may be attributed to two reasons. First, it is possible that American Twitter users do not explicitly mention the US when discussing the pandemic. Second, it is also conceivable that American Twitter users mention their state or city instead. Future work may look into these nuances further (Morstatter et al. 2013).

### Hate speech classification

We predicted hate speech scores for each tweet using a machine learning algorithm. We used a random forest classifier with handcrafted psycholinguistic features to enforce interpretability and scalability (Pennebaker et al. 2003; Tausczik and Pennebaker 2010). Using the Netmapper software, we extracted from each tweet several lexical measures of pronoun use, abusive words, exclusive words, absolutist words, and identity terms, among others (Carley et al. 2018b). Pairwise products of each linguistic measure were used as additional features to capture the ways they co-occurred within a tweet. Netmapper’s lexicon includes these measures for English as well as a variety of other languages including those used in the Philippines (e.g., Tagalog, Cebuano), thus facilitating our comparative analysis.

Our model was trained on a widely used benchmark dataset for hate speech (Davidson et al. 2017). This dataset distinguishes between hate speech, offensive speech, and regular speech as class labels. As previously mentioned, the difference between hate speech and offensive speech is crucial as it recognizes that some tweets may use expletives and similarly profane language but not hatefully target any group in particular. Our model achieved over 83% in terms of both accuracy and F1 score (Uyheng and Carley 2020a). Other experiments using alternative algorithms (e.g., logistic regression, support vector machines) consistently yielded results bested by random forest models. This gave us confidence in using our model for our purposes of analyzing the network dynamics of hate speech around COVID-19.

### Infodemic trends

Temporal analyses of these predictions further facilitated characterization of trends in hate speech spread over time. This analysis proceeds as follows: over the 75-day period under observation, we generated cumulative distributions of when each hateful tweet appeared in the dataset. Because of the probabilistic predictions generated by our machine learning model, we relied on three distinct cutoffs for classifying a tweet as hate speech. In order of increasing stringency, we relied on: (a) the median hate speech value in the dataset (i.e., the top 50% most hateful tweets), (b) the 75th percentile (i.e., the top 25% most hateful tweets), and (c) the tweets which achieved a hate speech probability of at least 50%.

Through this procedure, we produced three curves which visualize the infodemic spreading dynamics of hate speech in each country. Using the method described by Fisman and colleagues (Fisman et al. 2013), which was later adopted by Cinelli and colleagues for social media infodemics (Cinelli et al. 2020), we estimated a reproduction number $R_0$ which quantified the spread of hate speech within the online conversation. Also known as the Incidence Decay and Exponential Adjustment (IDEA) model, the Fisman model utilizes a fairly simple function to model the growth of an epidemic over time (Fisman et al. 2013). In particular, incidence *I* is modeled at time *t* (in days) with the reproduction number $R_0$ and some discounting factor *d* as follows:

$$\begin{aligned} I = \left( \dfrac{R_0}{(1+d)^t}\right) ^t. \end{aligned}$$

As in Cinelli et al. (2020), we let *I* represent the cumulative number of hateful tweets at time *t*. We estimate both $R_0$ and *d* through ordinary least squares regression. We note that Cinelli et al. (2020) estimate that $R_0$ on Twitter for COVID-19 infodemics lies in the confidence interval between 1.65 and 2.06. They additionally observe that using the more traditional SIR model resulted in unrealistic values of $R_0$ due to steep jumps in their dataset. We therefore adopt this insight in focusing on the IDEA estimate for $R_0$ in our analysis.

### Community detection

To examine the dynamics of groups in the online conversation, we represented our data as a time-varying social network. For our temporal analysis, we segmented the dataset into a series of daily snapshots. For a given day $t \in \left\{ 1,2,\ldots ,75\right\}$, let $G_t = (V_t, E_t)$ be the graph representation of the online conversation. Here, $V_t$ corresponds to the set of users in the data, represented as the set of vertices in the graph. Meanwhile, $E_t$ represents a set of weighted, directed edges between vertices in $V_t$. The weight of each directed edge is given by the number of interactions originating from the source node toward the target node. To obtain edge weights, we take the sum of all forms of Twitter communication, including retweets, replies, mentions, and quotes. The ORA software was used to perform all network analysis (Carley et al. 2018b).

Community detection was performed to operationalize a localized understanding of online groups. We used a Leiden algorithm to automatically recover local clusters of users. The Leiden algorithm is an unsupervised method for community detection which iteratively refines cluster assignments with the intuitive goal of optimizing the difference between actual and expected number of edges within an assigned cluster (Traag et al. 2019). It has been shown to be superior to the widely used Louvain algorithm by guaranteeing well-connected communities as well as faster runtime (Blondel et al. 2011). Thus, for each network snapshot, we obtained cluster assignments for all agents. Agents assigned to the same cluster were conceptualized as constituting a distinct group engaged in meaningful interaction about the pandemic. Note that for all succeeding analysis, we remove trivial clusters containing only one or two agents (i.e., isolates and pendants).

### Community-level hate metrics

Using the obtained groupings, we designed several novel measures for characterizing hate speech in its dynamic social context. Drawing on constructural theory, these metrics intuitively capture the aspects of the evolution of hate speech with online communities.

*Community-level hate.*

To obtain a continuous measure of hate-like content at a given time *t*, we leverage the probabilistic outputs of our random forest model. To propagate hate speech probabilities from tweets to users, we take each user’s average hate speech score at time *t*. As before, let $G_t = (V_t, E_t)$ represent the graph of the online conversation. Let $C_{t,1}, C_{t,2}, \ldots , C_{t,l_t}$ represent the $l_t$ distinct clusters derived by a Leiden algorithm. For each cluster $C_{t,i}$, where $i \in \left\{ 1,2,\ldots , l_t\right\}$, consider its constituent agents $V_{t,i}$. Now let $h_{t,i,j}$ represent the hate score associated with user $j \in V_{t,i}$. Then the cluster-level hate $H_{t,i}$ is given by the average of user-level hate speech scores, as given by the following equation:

$$\begin{aligned} H_{t,i} = \dfrac{1}{|V_{t,i}|}\sum \limits _{j \in V_{t,i}} h_{t,i,j}. \end{aligned}$$

*Hate community homogeneity.* Beyond the raw amount of hate in the network, we are also interested in the extent to which users employing more hate speech are in turn more likely to interact with more hateful others. This would also correspond to hate speech being more organized and less scattered throughout the online conversation at a given time. We refer to this measure as *hate community homogeneity*.

As above, let $\sigma _{t,i}^2$ represent the variance of user-level hate speech scores $h_{t,i,j}$ within the same cluster *i*. Then we also obtain a measure $O_{t,i}$ of how homogeneous or orderly the cluster-level hate speech is. Higher levels of this measure indicate that hateful users are conversing with other hateful users. Lower levels suggest that hateful users are more scattered throughout the social network, interacting with both hateful and non-hateful users.

At a given time *t*, we compute hate community homogeneity for each cluster *i*. Taking the measurement in log scale deals with differences in scale (especially extremely small values), with arbitrarily small $\nu > 0$ ensuring all inputs are non-zero. The equation is given as follows:

$$\begin{aligned} O_{t,i} = \log \biggr [ \dfrac{H_{t,i}}{\sigma _{t,i}^2} + \nu \biggr ]. \end{aligned}$$

Finally, at time *t*, we obtain an overall network-level measure of hate community homogeneity $O_t$ using an average of cluster-level hate community homogeneity scores as follows:

$$\begin{aligned} O_{t} = \dfrac{1}{l_t}\sum \limits _{i = 1}^{l_t} O_{t,i}. \end{aligned}$$

*Hate speech assortativity.* Leveraging more classical network science measures, we also use the assortativity coefficient to analyze the relational dynamics of hate speech over time. Newman defines assortativity as the tendency for nodes in a network to be connected to similar nodes (Newman 2003). For each time *t*, we measure the assortativity of network $G_t$ based on node-level hate speech scores $h_{t,i,j}$ as defined above. In this manner, we obtain another measure of the organization of hate speech over time. For continuous variables, the assortativity coefficient is given simply by the Pearson correlation between the user-level hate speech scores of source nodes with the user-level hate speech scores of target nodes. We recall that $G_t$ is a directed network, so these values are not symmetric.

While our proposed hate community homogeneity measure depends on clusters derived through a Leiden algorithm, the assortativity coefficient does not depend on network clustering. Hence, while both network measures capture some notion of hate speech organization in the network, they are not equivalent. Hate community homogeneity accounts for a wider context of social influence than assortativity; the latter, meanwhile, focuses primarily on dyadic interaction. However, both values considered together may nonetheless be informative for analysis, as we demonstrate later.

*Structural features of hate communities.*

Next, we consider the structural features of clusters, following the hypothesis that these relate to localized levels of hate speech (Kim 2020). We are specifically interested in the following features. First, we examine cluster size, denoted by the number of unique agents assigned to the same cluster. Second, we look at the E/I index. The E/I index is a classical measure in network science which intuitively quantifies exclusive group communication (Krackhardt and Stern 1988). Normalized between +1 and − 1, higher values of the E/I index indicates high levels of communication with out-groups; lower levels suggest that the cluster communicates solely with in-group members. Third, we measure the Cheeger constant. This quantifies bottleneck behavior, such that higher values indicate more hierarchy in the cluster while lower values indicate more dispersed connection patterns between agents (Mohar 1989).

*Identity target analysis.*

To analyze the content and targets of hate speech, we employ a lexicon of identity terms derived from prior research (Joseph et al. 2014, 2016). Identity terms here refer to words which describe personal or group-based categories (Priante et al. 2016). Available on the Netmapper software (Carley et al. 2018b), our analysis recognizes that identities may be intersectional; hence, identity terms are further organized into subcategories of gender, race/ethnicity, politics, and religion.

Each of these subcategories is counted over each tweet using the Netmapper software. User-level invocation of identity terms is computed as the average number of times an account mentions each identity subcategory in their tweets at a given time. Cluster-level invocation of identity terms is computed as the average user-level identity score for all accounts within the cluster.

To capture the sense that some identities are targeted over others in hate speech, we compute the linear slope relating the level of cluster-level hate speech to cluster-level identity scores. We use ordinary least-squares regression to compute these slopes for a given time interval, thereby reflecting the extent to which certain identity categories are more or less associated with hate speech within a particular time frame.

### Integrated estimation of network dynamics

Finally, we present an integrated analysis of structural (i.e., cluster properties) and functional (i.e., identity targets) network dynamics of hate speech. Invoking insights from constructural theory, we posit that levels of cluster-level hate are dynamically associated with: (a) cluster-level measures of information flow, and (b) cluster-level lexical measures linked to identity targets of hate speech.

We model these intuitions in a Bayesian multiple regression setup. In this model, we consider daily clusters as the unit of analysis. For each cluster at a given point in time, we consider the fixed effects of structural features (i.e., cluster size, density, E/I index, and Cheeger score) and functional features (i.e., identity scores) of hate speech. To deal with the temporal component of our analysis, we model an AR1 random effect. The dependent variable is given by the cluster-level hate. Uninformative, standard Gaussian priors are used for all estimated effects.

To perform model inference, we use the Integrated Nested Laplace Approximation (INLA) technique (Rue et al. 2009). INLA is a fast alternative to traditional techniques like Markov Chain Monte Carlo estimation methods. INLA retrieves high-quality estimations of model parameters at scalable runtime through the use of appropriate Gaussian approximations of general distributions. This allows us to efficiently analyze our relatively large-scale dataset and estimate the effects of both structural and functional features of hate speech networks over time. The additional advantage of our Bayesian approach is that we also obtain interval estimates of uncertainty instead of single point-estimates.

## Results

Our analysis reveals idiosyncratic network dynamics associated with the spread and organization of hate speech surrounding the COVID-19 pandemic. In the succeeding sections, we first trace temporal trends in community-level hate speech for both the US and the Philippines. We also consider the associated infodemiological characteristics of hate speech spread and compare it to known social media infodemics around COVID-19. We then examine patterns in the identity-based targets of hate communities. Finally, we present our integrated analysis of how structural and functional features of dynamic network clusters relate to community hate.

### Hate communities over time

**Fig. 1:**
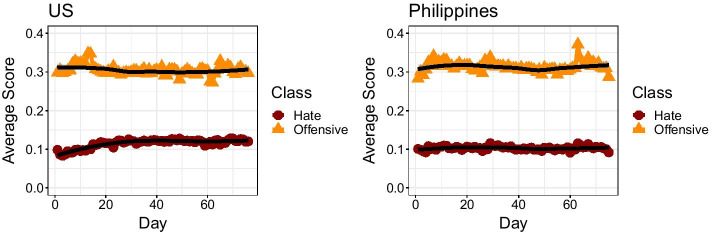
Average community-level scores on hate speech and offensive speech over time. Smoothed loess trends are also depicted. We observe relative stability in average scores for both types of speech, with offensive speech consistently higher throughout the time period under examination

Hate speech predictions in both datasets suggest a relatively consistent average level of online hate over time. Figure 1 depicts temporal trends in average community-level hate speech, with predicted offensive speech levels as a point of comparison. In general, hate speech levels were relatively low compared to regular speech and offensive speech. Interestingly, similar levels of both hate speech and offensive speech were detected across the two contexts under study. Furthermore, while noticeable spikes may be observed, the average hate speech score period did not go beyond 0.2 in either the Philippines or the US. That said, while the Philippine trend remained fairly stable over time, a modest but consistent increase was observed in the US trend.

Our datasets comprise primarily mainstream hashtags. Based solely on general community-level patterns of hate speech prevalence, we do see that a non-negligible proportion of the online COVID-19 conversation pertained to inappropriate content. That being said, the online conversation remained dominated by more general topics around the disease and not overt hate.

**Fig. 2:**
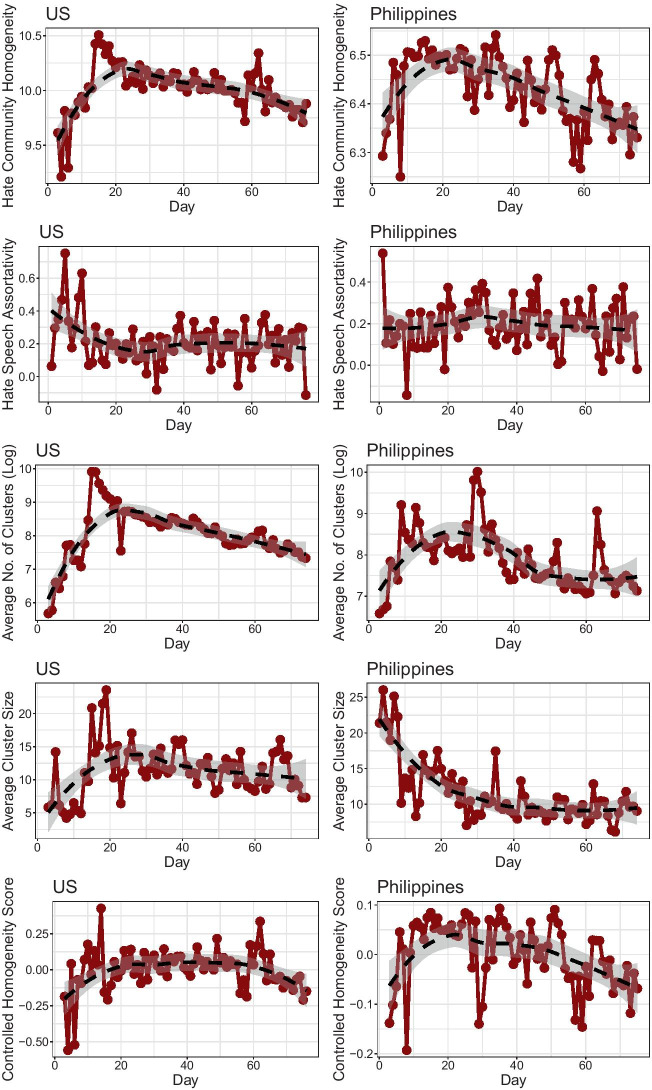
Network measurements of hate speech organization over time with 95% smoothed loess trends. **Row 1:** Hate community homogeneity measures. **Row 2** Hate speech assortativity measures. **Row 3:** Average number of Leiden clusters. **Row 4:** Average cluster sizes. **Row 5:** Hate homogeneity scores controlling for assortativity, average number of clusters, and average cluster size. Overall analysis suggests robust pattern of initial increase in community-level but not dyad-level organization of hate communities, followed by their gradual dissipation over time

Community-level organization of hate speech, however, suggests a more nuanced picture. Here, we observe a distinct pattern in the extent to which hate communities organize over time. The top row of Fig. 2 suggests that within the first twenty days captured in the dataset—over the entire month of March—the average community homogeneity in hate speech levels rapidly increased for both countries. This is in contrast to the more conventional assortativity measure in network science presented in the second row of Fig. 2, which focuses solely on dyadic interactions (Newman 2003). In fact, we noticed that in the US, the correlation between hate community homogeneity and hate speech assortativity was negative ($r = -0.3288, p < .01$); while it was non-significant in the Philippines ($r = 0.0342, p > .05$). For more robust analysis, we also present temporal trends in the average number of clusters derived by Leiden clustering in the third row of Fig. 2, as well as the average size of these clusters in the fourth row. Controlling for these two factors alongside hate speech assortativity, we additionally plot a controlled community homogeneity score in the fifth row that robustly retains the general pattern observed.

In other words, while the average level of hate did not itself increase much over time, COVID-19 conversations in March saw more hateful users cluster together, pointing to the formation of more coherent hate communities. Because this measure focuses on cluster-level connections instead of direct interactions as captured by the assortativity coefficient, this suggests the significance of network clusters as a unit of analysis in considering the spread of hate speech. In other words, at time points featuring high levels of hate community homogeneity but low assortativity, hateful accounts may themselves not interact with each other. But it is possible, for instance, that multiple hateful accounts may be targeting the same non-hateful account, as in coordinated harassment campaigns; or a single hateful account may be targeting multiple non-hateful accounts, as in the actions of a hateful opinion leader or an influential troll (Uyheng and Carley 2020a).

Moreover, while this temporal pattern in hate community homogeneity was common between both the US and the Philippines, the US saw a significantly higher range of homogeneity scores overall. Here, homogeneity scores ranged between 9 and 10.5. Meanwhile, in the Philippines, values ranged between 6 and 7. As defined, the community homogeneity score is driven by both the mean and the variance of community-level hate speech. However, since we know that average community-level hate speech scores were similar across the two datasets, we may infer that hate speech tended to feature lower variance—that is, it was more organized—on a community level in the US than in the Philippines.

In the months that followed, hate community homogeneity steadily decreased. Hateful users thus grew more dispersed throughout the social network. In other words, more hateful users were interacting with less hateful users, instead of primarily interacting amongst themselves. Nevertheless, even in this period of decline, the US still consistently saw higher hate community homogeneity scores than the Philippines. Moreover, decreases in homogeneity resulted back to early March levels for the Philippines, while homogeneity in the US decreased less dramatically. This further indicates that hate communities were also more stable in the US than in the Philippines.

### Infodemiological characteristics of online hate speech

**Fig. 3:**
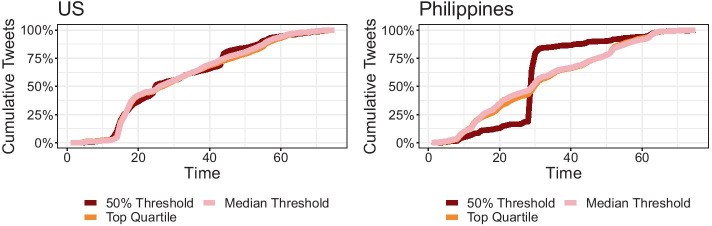
Cumulative generation of hateful tweets following different thresholds for hate speech classification. Hateful tweets in the US generally follow similar trends in the US regardless of classification type. The Philippines features a noticeable spike in hateful tweets when using the strictest threshold

Infodemiological analysis offers additional insight into these dynamics. Here, we observe the cumulative generation of hateful tweets in Fig. 3. Using three different thresholds for hate speech classification offers some insight into how more hateful (or less hateful) tweets may be generated at different rates.

In the US, we see fairly consistent infodemiological trends, whereby accounts belonging to all defined user-level hate scores feature similar arrival rates. For all tweets classified as hate speech, however, we see that the cumulative generation plot curves upward approaching the 20th day in our dataset, around the time that the hate community homogeneity is highest. Before then, the curve is generally much flatter. Hence, it appears that a large number of high-hate tweets—regardless of classification threshold—are incorporated into the conversation at the point where hate communities are most well-organized.

Meanwhile, in the Philippines, we may observe a similar pattern when using the less stringent thresholds (i.e., median hate score and top quartile). However, we also observe a distinct spike in hate speech generation when using the strictest probability threshold. For most of March, the extremely hateful tweets are generated at a much slower rate. However, an inflection point is observed at the end of the month, during which hate speech is produced at a significant rate, accounting for majority of the most hateful tweets. Toward the end of April, we see that the most hateful tweets have already been generated and continue to be produced at a steadier pace. This more steady generation of hate speech coincides with the decline in hate community homogeneity, or the dispersal of hateful accounts across the social network. Hence, we may infer that in the Philippine case—as in the US case—the highest point of hate speech generation is at the point of highest hate community homogeneity. In other words, high levels of organization of hate communities coincided with high proliferation of hateful content in COVID-19 conversations.

Based on these cumulative generation curves, we derive an infodemiological analysis of online hate speech proliferation around COVID-19, as summarized in Table 1. Three things are notable from this analysis. First, as a more stringent classification threshold is used, a lower $R_0$ value is detected. This is expected as more stringent classifications result in lower estimates of hate speech proliferation. Second, we observe that all values are close to the range of 1.65–2.06 found by Cinelli and colleagues regarding the spread of information around COVID-19 on Twitter (Cinelli et al. 2020). Hence, it appears that although hate speech features at a lower volume than other COVID-19 information, its spreading rates are comparable to other COVID-19 information.

**Table 1:** Estimated reproduction number (and dampening factors) of online hate speech using the IDEA model described by Fisman et al. (2013) and adapted by Cinelli et al. (2020) for social media infodemics

Country	Threshold	$R_0$	*d*
US	50% threshold	1.4380	0.0036
	Top quartile	2.0421	0.0075
	Median threshold	2.1120	0.0079
Philippines	50% threshold	1.5069	0.0041
	Top quartile	2.1061	0.0079
	Median threshold	2.1593	0.0082

Similar levels are detected for both the US and the Philippines across different hate speech classification thresholds

### Community targets of hate speech

**Fig. 4:**
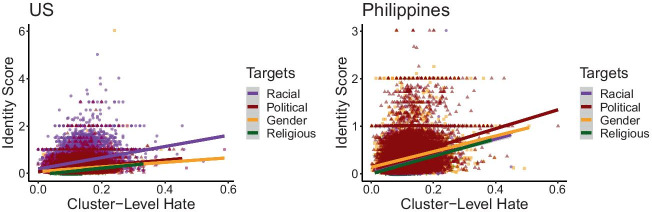
Overall distribution of identity targets relative to cluster-level hate. Linear trends are also depicted for each identity category, visualizing the overall association between hate speech and identity groups across all time periods. Between the two countries, distinct patterns in rank order are observed across the identity categories, with higher hate associated with racial identities in the US and higher hate associated with political identities in the Philippines

Analysis of the content of hate speech reveals further insights into the identities targeted across both settings. Figure 4 shows how different identity categories are associated with community-level hate across the US and the Philippines. In general, higher levels of hate are positively associated with more frequent invocations of identity subcategories. This is consistent with our definition of hate speech as targeted negative talk.

However, we notice that in the US, the slope is steepest for racial identities. This indicates that across all time periods, hate speech is most directed toward racial identities. On the other hand, we observe that political identities have the steepest slope in the Philippines. By contrast, we observe that racial hate in the Philippines appears to have among the flattest slopes. This suggests key differences in the content of hate speech for the two countries.

**Fig. 5:**
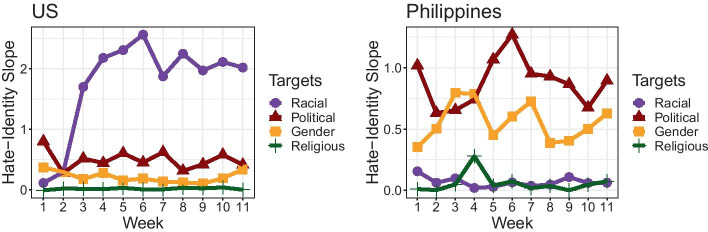
Weekly variations in hate-identity associations. Values depict the estimated slope of the OLS regression line between cluster-level hate and cluster-level identity scores for each week. Distinct patterns are observed between the two countries, suggesting different temporal trends in the identities targeted by the hate communities

These differences are further borne out over time. Figure 5 shows the ways that hate-identity associations fluctuate on a weekly basis. In the US, racial identities remain the predominant target of hate speech over all weeks under observation, except for the first 2 weeks, where hate appears more directed toward political identities. On the other hand, we note that hate in the Philippines is also dominated by political identities. Weeks 3 and 4 briefly show how gendered hate overtakes political hate, but for the remaining time, political hate remains dominant.

### Network dynamics of hate speech

**Fig. 6:**
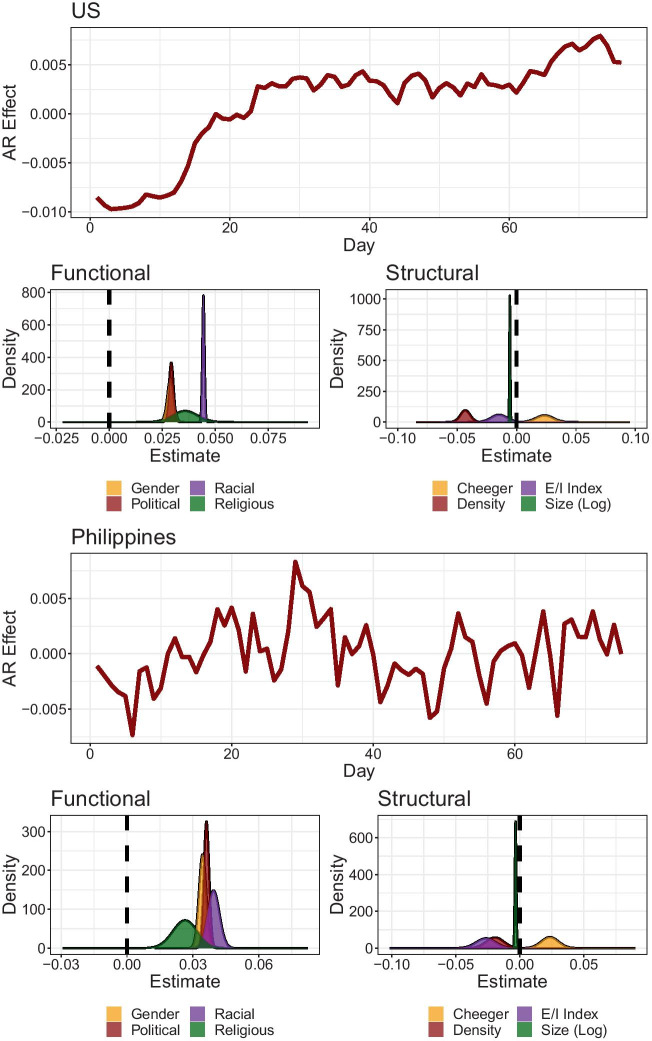
Estimated marginal distributions for structural and functional cluster features of hate communities obtained using INLA. Temporal random effects are modelled as AR1. Functional and structural features of hate communities depicted as Gaussian distributions. The vertical line marks the zero point, indicating no average effect. Distinct temporal and functional characteristics are detected between the two countries, but structural dynamics are consistent

Finally, we analyze the results of model inference through INLA. Figure 6 shows the marginal distributions of the structural and functional cluster features predictive of community-level hate speech. We also observe temporal random effects in both countries, which reflects a steady increase in the US versus more fluctuations in the Philippines.

In both countries, we observe consistent directions in the effects of both structural and functional features. Higher levels of community-level hate were consistently predicted by clusters having smaller size (US: $b_{mean} = -0.006, SD = 0.000$; PH: $b_{mean} = -0.003, SD = 0.001$), featuring lower E/I index (US: $b_{mean} = -0.015, SD = 0.007$; PH: $b_{mean} = -0.026, SD = 0.007$), lower density (US: $b_{mean} = -0.043, SD = 0.004$; PH: $b_{mean} = -0.019, SD = 0.009$), and higher Cheeger scores (US: $b_{mean} = 0.023, SD = 0.007$; PH: $b_{mean} = 0.023, SD = 0.007$). Collectively, these suggest that smaller communities featuring more isolated and hierarchical interactions are more likely to feature hate over time.

We also see that all identity categories are positively associated with higher levels of community hate. Higher community-level invocation of racial (US: $b_{mean} = 0.044, SD = 0.001$; PH: $b_{mean} = 0.039, SD = 0.003$), political (US: $b_{mean} = 0.029, SD = 0.001$; PH: $b_{mean} = 0.036, SD = 0.001$), gender (US: $b_{mean} = 0.029, SD = 0.001$; PH: $b_{mean} = 0.034, SD = 0.002$), and religious (US: $b_{mean} = 0.036, SD = 0.006$; PH: $b_{mean} = 0.026, SD = 0.006$) identities all had marginal distributions concentrated on positive values. In the US, as expected, we saw that the mean value of the race distribution had the highest value, with very little variance. This indicates the extremely charged nature of racialized discourse in the US COVID-19 conversation. Interestingly, we also saw that the mean of the race distribution had the highest value in the Philippines, despite prior analysis which points to the primacy of political identities. While the political distribution is also concentrated on high values, this observation indicates that when other identities are controlled for, race still matters in online hate speech in the Philippines.

## Discussion

As the world continues to wrestle with the COVID-19 pandemic, as a social issue going beyond public health, our work sheds light on important dynamics underlying online hate (Van Bavel et al. 2020). Our findings suggest that while most online hate remains relatively rare in the overall conversation, its spread can be highly volatile—even comparable to other viral COVID-19 information (Cinelli et al. 2020)—and this proliferation depends crucially on the behaviors of online communities (Johnson et al. 2019; Kim 2020). More specifically, we find that hate thrives in smaller, more isolated, and more hierarchical groups. This resonates with concerns regarding interactions within echo chambers and sequestered communities, whereby individuals begin to participate away from the mainstream of public discourse, and potentially engage in more harmful online behaviors (Carley et al. 2018a; Geschke et al. 2019; Uyheng and Carley 2020b).

Beyond the structural features of hateful communities, we also find that the content of hate matters. While all identities are positively associated with increased community-level hate, race and politics feature as the crucial drivers of hate in the US and the Philippines. As shown in prior work (Stechemesser et al. 2020; Ziems et al. 2020), the issue of racism grew salient in online conversations surrounding the pandemic. Prejudicial, sinophobic talk may become a way for political leaders and their followers to oversimplify and make sense of the pandemic (Reicher and Stott 2020). Yet such processes are tied up with broader, longer-term concerns over geopolitical relations, with strained US-China relations and Philippine-China relations potentially contributing to a climate ripe for the spread of anti-China sentiments (Cohen 2020; Montiel et al. 2019; Ong et al. 2019).

Furthermore, politically directed hate likewise played a central role, particularly in the Philippine conversation. Here, public health concerns were compounded with additional political challenges of a polarized society and fragmented government response to the pandemic (Montiel and Uyheng 2020; Uyheng and Montiel 2020b). Hate, in this case, does not ‘strike down’ toward any racial minority, but ‘strikes up’ at political leaders deemed ineffective at addressing the public health crisis (Lasco 2020; Uyheng and Montiel 2020a). While both forms of hate thus operate with similar community structures, it is pertinent to recognize their distinct functions, and contributions to wider societal strain. Addressing these issues from a policy perspective, for instance, will vitally require this nuanced distinction (Burnap and Williams 2015; Carley et al. 2018a; Jardine 2019).

Taken together, our work points to the conceptual utility of a constructural lens, with its empirical application of network methods, to analyze the community dynamics of hate speech beyond its textual prevalence (Carley 1989, 1991, 1995). Especially viewed over time, network dynamics offer a powerful, general lens with which to view universal as well as context-specific features of diverse social phenomena. Methodologically, we especially highlight the value of examining network features from multiple levels, such as through analysis of both dyadic assortativity and broader cluster-level homogeneity of hate speech measures (Newman 2003). Blended with an infodemiological approach, our dynamic network framework offered a quantitative portrait of how online toxicity may proliferate around online COVID-19 conversations in two distinct political and cultural settings. These methods may be extended to cover similar phenomena related to information spread on social networks, both during the pandemic and beyond (Cinelli et al. 2020; Gallotti et al. 2020; Zarocostas 2020).

These contributions notwithstanding, we also point to several limitations in the work presented here. First, sampling constitutes an important constraint on Twitter research at large, as it is difficult to extrapolate data collected using the API to the total conversation (Morstatter et al. 2013). In relation to this, we also posit that different levels of hate—and potentially different spreading dynamics—may be observed with data collected using more detailed search terms (e.g., state-level keywords), or more explicitly toxic search terms as in related work (Ziems et al. 2020). Furthermore, while we valued interpretability and scalability of methods in our analytical pipeline (MacAvaney et al. 2019), we also acknowledge that advances in natural language processing and machine learning in general may enable more cutting-edge feature enrichment for the analysis presented here (Badjatiya et al. 2017; Fortuna and Nunes 2018).

Future work may thus build on these gaps in extending our findings. It may be fruitful, for instance, to test whether the structural and functional features of hate speech determined here apply to broader datasets (e.g., COVID-19 tweets in general) or other nationally defined datasets (e.g., contexts beyond the US and the Philippines). It may also be of interest to relate the spread of online hate speech to the real-world spread of COVID-19 (Gallotti et al. 2020; Hâncean et al. 2020). Conversely, more fine-grained psychological processes could also be examined in relation to the findings we present here, which only measure the communication of hate, but do not explicitly measure cognitive or emotional shifts. Methodological advances on any of the algorithms mentioned here would likewise provide more precise estimates of hate and its associated community dynamics.

## Data Availability

The datasets and analysis code used in the current study will be made available on the Kilthub repository upon acceptance. Further details on both ORA and Netmapper software are provided at https://netanomics.com/.

## Abbreviations

COVID-19: Coronavirus Disease 2019
US: United States
API: Application programming interface
URL: Uniform resource locator
JSON: JavaScript object notation
IDEA: Incidence Decay and Exponential Adjustment
ORA: Organization Risk Analyzer
INLA: Integrated Nested Laplace Approximation
SD: Standard deviation
AR1: Autoregressive Model with Lag 1
